# Combined effect of prefrontal transcranial direct current stimulation and a working memory task on heart rate variability

**DOI:** 10.1371/journal.pone.0181833

**Published:** 2017-08-03

**Authors:** Stevan Nikolin, Tjeerd W. Boonstra, Colleen K. Loo, Donel Martin

**Affiliations:** 1 School of Psychiatry, University of New South Wales, Black Dog Institute, Sydney, Australia; 2 Systems Neuroscience Group, QIMR Berghofer Medical Research Institute, Brisbane, Australia; 3 St. George Hospital, Sydney, Australia; University Medical Center Goettingen, GERMANY

## Abstract

Prefrontal cortex activity has been associated with changes to heart rate variability (HRV) via mediation of the cortico-subcortical pathways that regulate the parasympathetic and sympathetic branches of the autonomic nervous system. Changes in HRV due to altered prefrontal cortex functioning can be predicted using the neurovisceral integration model, which suggests that prefrontal hyperactivity increases parasympathetic tone and decreases contributions from the sympathetic nervous system. Working memory (WM) tasks and transcranial direct current stimulation (tDCS) have been used independently to modulate brain activity demonstrating changes to HRV in agreement with the model. We investigated the combined effects of prefrontal tDCS and a WM task on HRV. Bifrontal tDCS was administered for 15 minutes at 2mA to 20 participants in a sham controlled, single-blind study using parallel groups. A WM task was completed by participants at three time points; pre-, during-, and post-tDCS, with resting state data collected at similar times. Frequency-domain HRV was computed for high frequency (HF; 0.15–0.4Hz) and low frequency (LF; 0.04–0.15Hz) power reflecting parasympathetic and sympathetic branch activity, respectively. Response time on the WM task, but not accuracy, improved from baseline to during-tDCS and post-tDCS with sham, but not active, stimulation. HF-HRV was significantly increased in the active tDCS group compared to sham, lasting beyond cessation of stimulation. Additionally, HF-HRV showed a task-related reduction in power during performance on the WM task. Changes in LF-HRV were moderately inversely correlated (r > 0.4) with changes in WM accuracy during and following tDCS compared to baseline levels. Stimulation of the prefrontal cortex resulted in changes to the parasympathetic branch of the nervous system in agreement with a linearly additive interpretation of effects. Sympathetic activity was not directly altered by tDCS, but was correlated with changes in WM performance. This suggests that the parasympathetic and sympathetic branches respond differentially due to similar, but distinct neural pathways. Given the ease of HRV data collection, studies of prefrontal tDCS would benefit from collection of this data as it provides unique insight into tDCS effects resulting from propagation through brain networks.

## Introduction

Heart rate variability (HRV), an index of cardiac adaptation to allostatic load, is known to be regulated by the prefrontal cortex such that changes in prefrontal cortex functioning show measurable effects on HRV [1]. Alteration of prefrontal cortex activity has been independently demonstrated to modulate HRV using both non-invasive brain stimulation [2], and cognitive tasks reliant on prefrontal functioning [3]. However, several outstanding questions remain. Specifically, whether brain stimulation of the prefrontal cortex alters HRV at rest, whether these effects outlast the period of stimulation, and what the combined effect of both prefrontal cortex brain stimulation and a task known to predominantly engage regions of the prefrontal cortex is on the autonomic nervous system.

**Fig 1:**
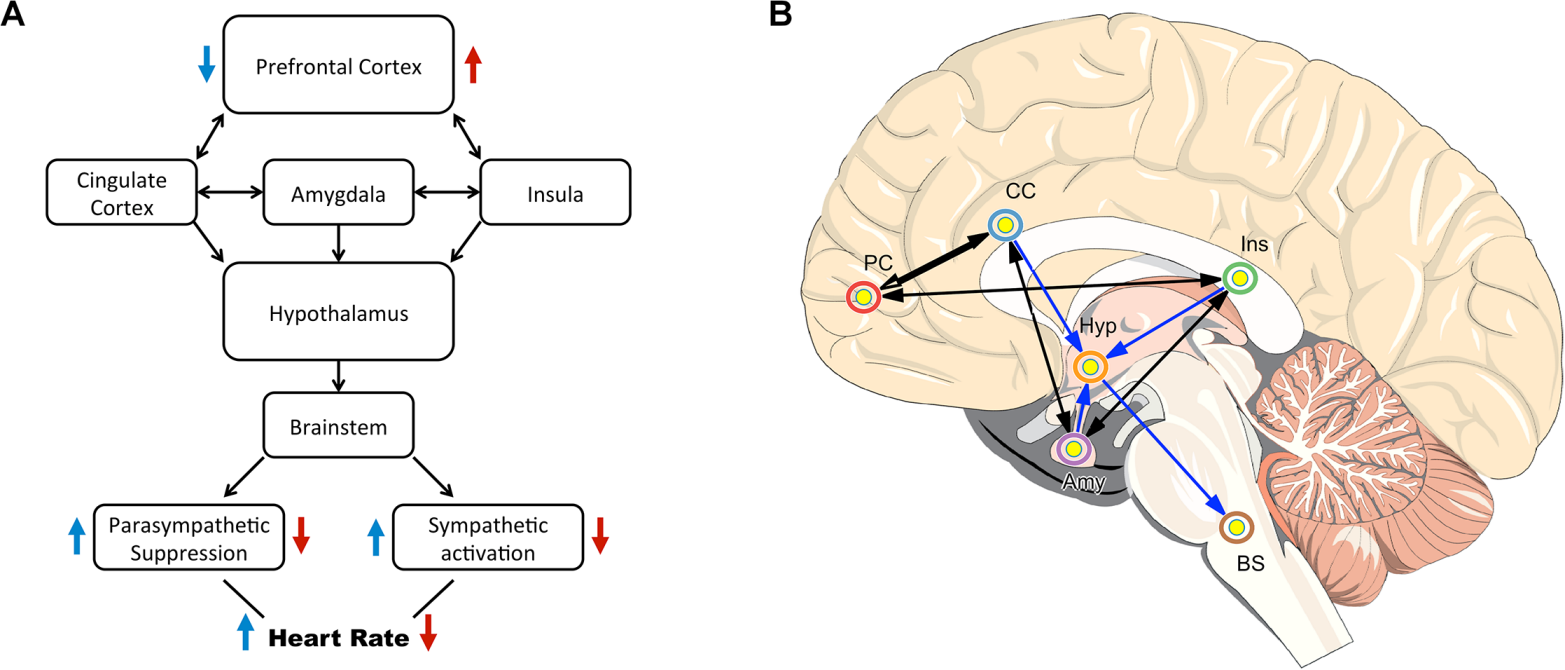
Neurovisceral integration model. (A) Simplified depiction of the neurovisceral integration model described by Thayer and Sternberg [1]. (B) Brain regions relevant to the neurovisceral integration model. PC, prefrontal cortex; CC, cingulate cortex; Hyp, hypothalamus; Ins, insula; Amy, amygdala; BS, brainstem.

The prefrontal cortex is known to modulate brain regions involved in the regulation of autonomic nervous system activity, such as heart rate [4]. Both parasympathetic and sympathetic branches of the nervous system are mediated by cortical-subcortical pathways which involve the prefrontal cortex, the anterior cingulate cortex, the insula, the hypothalamus, and the brainstem [5]. The neurovisceral integration model posits that the prefrontal cortex regulates and tonically inhibits activity in limbic structures which act to supress parasympathetic activity and activate sympathetic circuits–see Fig 1 [1]. Variation in the output of these two branches of the autonomic system produces heart rate variability (HRV), a measure of autonomic nervous functioning [6]. Therefore, activation of the prefrontal cortex results in change to HRV [7,8], which can be thought of as a measure of the aggregate effect of activity in a complex brain network, regulated top-down by the prefrontal cortex. According to the model, hyper-activation of the prefrontal cortex inhibits the sympathoexcitatory circuit of the amygdala, which is known to have outputs relevant to autonomic regulation [1]. This in turn reduces sympathetic activity and parasympathetic suppression, culminating in a reduction in heart rate.

Evidence in support of this model has come from studies which have moderated activity within the prefrontal cortex using cognitive tasks [1,9,10], as well as non-invasive brain stimulation [2], and measured autonomic nervous system activity using HRV as a marker. For example, Gianaros et al. [11] found evidence for associations between working memory task difficulty, corresponding decreases in vagally mediated HRV, and a concurrent change in regional cerebral blood flow (rCBF) in the medial prefrontal cortex. In addition, a meta-analysis of the relationship between cognitive and emotional processes and HRV found significant associations in key areas such as the amygdala and the ventromedial prefrontal cortex via changes in rCBF [12].

The effects of moderating prefrontal function have also been studied using non-invasive brain stimulation. Transcranial direct current stimulation (tDCS) is a form of non-invasive brain stimulation which modulates neuronal functioning, resulting in both excitation and inhibition of neuronal activity at regions of interest that lie between the electrodes [13,14]. The dorsolateral prefrontal cortex (DLPFC), an important node in the fronto-parietal network [15], has been the focus of much tDCS research due to its relevance for psychiatric illnesses [16–18], and its role in subserving higher level cognitive functions, including working memory [19,20]. Brunoni et al. [2] investigated the effects of anodal tDCS to the left DLPFC during performance on an emotional picture viewing task, finding increased vagal activation, as measured using the power spectra of heart beat intervals within the high frequency (HF) range (0.15–0.4Hz). However, to-date no study has investigated the effects on HRV of prefrontal tDCS under resting state conditions, though the neurovisceral model would predict that increased activation of the prefrontal cortex leads to an increased parasympathetic and decreased sympathetic output.

HRV has been used to examine task-induced cognitive stress, demonstrating an inverse relationship between working memory load and sympathetic activity i.e. greater demands on working memory, which require higher levels of operator effort, suppress output to the sympathetic branch of the nervous system [21]. Furthermore, working memory load has been correlated with prefrontal connectivity [22], suggesting this outcome is in agreement with the neurovisceral integration model, which predicts a reduction in tonic suppression of the parasympathetic branch of the autonomic nervous system, thereby increasing vagal tone, whilst simultaneously decreasing sympathetic activity [1]. TDCS, when administered to the prefrontal cortex during a working memory task, has been shown to augment performance [23,24]. Thus, the facilitatory effects of prefrontal tDCS during a working memory task could be expected to decrease the relative mental load placed on the participant, resulting in performance enhancement as well as mitigation of the cognitive stress-related inhibitory effect on sympathetic activity. Furthermore, the neurovisceral model predicts a positive relationship between parasympathetic tone and performance on the cognitive task due to increased prefrontal activation.

Here we investigate changes in HRV induced by prefrontal tDCS and a working memory task subserved by the left DLPFC using the neurovisceral integration model as a theoretical framework to interpret results. Using the model, stimulation of the left DLPFC using only tDCS is expected to increase parasympathetic tone and diminish sympathetic activity relative to sham. Similarly, improvements in cognition mediated by prefrontal regions are anticipated to correlate with HRV markers of parasympathetic and sympathetic branches (positively and inversely, respectively). Finally, these changes should continue beyond stimulation due to the lasting neuroplastic effects of tDCS, which have been reported to last up to an hour [25,26].

## Materials and method

### Participants

Twenty healthy participants were randomly allocated to receive either active or sham tDCS. All participants provided informed consent prior to participation. Recruitment was achieved through a university website, and thus attracted predominantly students (mean age: 22.8 ± 3.5; range: 18–30). Exclusion criteria included significant psychological or neurological illness, excessive alcohol or illicit substance abuse, smoking, and ambidextrous or left-handed applicants assessed using the Edinburgh handedness test [27]. The experimental protocol was approved by the UNSW Human Research Ethics Committee (HC13278) and all participants gave written informed consent prior to participation. This study forms a subset of a larger study of the effects of tDCS on cognitive performance using concurrent EEG.

### Protocol

To investigate the effect of prefrontal tDCS on HRV and the relationship with cognitive functioning, we acquired electrocardiography (ECG) before, during and after tDCS to the left DLPFC while participants were at rest or performing a working memory task. Participants were comfortably seated in a chair in a partially soundproofed room and placed in front of a computer screen positioned at approximately eye level. The working memory task we used was a visual 3-back task, which was adapted from Mull and Seyal [28]. The task required participants to press a key when the letter displayed on the screen matched the letter presented three trials previously. Participants were first given the opportunity to practice the task for five minutes, and were then stratified according to their performance on a second five minute long presentation of the same task. Stratification was achieved using d-prime (d’) scores obtained from a similar experiment of working memory in healthy participants [23] into low (1.5 ≤ d’ score < 2.5), medium (2.5 ≤ d’ score < 3.5), and high (3.5 ≤ d’ score) performance categories. Following stratification, participants were randomised to receive either active or sham tDCS. Inquisit 4 (Version 4, Millisecond Software) was used to administer the working memory task and to record button presses.

**Fig 2:**
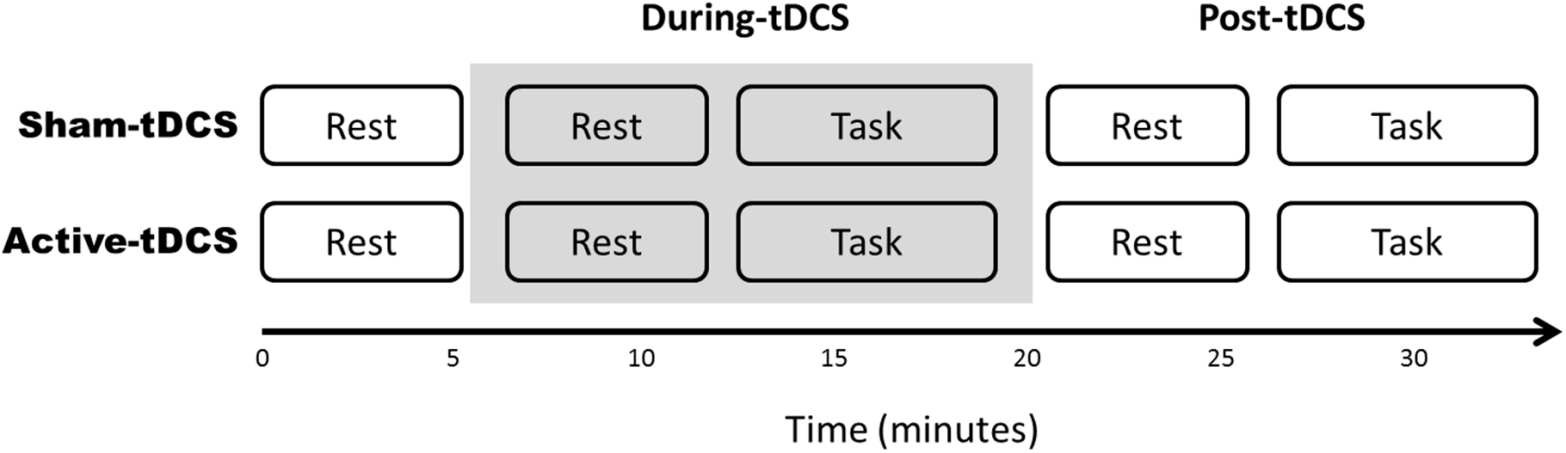
Study design using parallel groups. HRV data was collected at five epochs, each lasting five minutes. ECG data were recorded at periods of rest occurring at baseline, as well as during-tDCS and post-tDCS, in addition to task-related activity during-tDCS and post-tDCS. Shaded block indicates period during which tDCS was administered.

Following stratification, setup of electrodes used for ECG, electroencephalography (EEG) and tDCS took approximately 30 minutes. The experiment itself took a further 35 minutes to complete and ECG was acquired for the entire duration. A baseline recording was first obtained for five minutes at rest. Participants then received tDCS for 15 minutes, during which they sat at rest for the first five minutes then performed a working memory task for an additional seven minutes. Following tDCS, participants were again at rest for five minutes prior to completing a working memory task again. Resting state activity was recorded with eyes open and focussed on a fixation cross presented on the screen. The order of tasks is displayed in Fig 2.

### Transcranial direct current stimulation

TDCS was delivered using an Eldith DC stimulator (Neuroconn, Illmenau, Germany) for 15 minutes. A current intensity of 2mA was applied through saline-soaked sponge electrodes measuring 4cm x 4cm (area = 16cm^2^) resulting in a current density of 1.25Am^-2^. Participants received bifrontal tDCS with the anode placed over the left DLFPC (F3 according to International 10–20 system), and the cathode located over the right DLPFC (F4). Sham stimulation was gradually ramped up to 2mA over 30s, maintained in intensity for 30s to elicit paraesthetic sensation and preserve participant blinding [29], and then ramped back down over a further 30s.

### ECG data acquisition

ECG data was obtained as part of an EEG recording set up using a 72-channel TMSi Refa amplifier (TMS International, Oldenzaal, Netherlands). Two Ag/AgCl electrodes were placed below the right clavicle and on the lower left ribs to capture ECG. Data was sampled at 2048Hz and stored to disk.

### Data analysis

QRS complexes, the combinations of electrical waves representing ventricular depolarisation, were identified in the ECG data using custom developed code on Matlab R2014b (the MathWorks, Inc., USA). R-R time intervals were extracted and processed in Kubios HRV V2.1 software [30]. HRV power spectra were calculated using non-parametric, Fourier-based methods. Piecewise cubic spline interpolation was employed to remove artefacts and ectopic beats with an interpolation rate of 4Hz.

ECG recording was conducted throughout the whole experiment and segmented offline into 5 min epochs corresponding to resting-state recordings and to task-related activity during the working memory task. Task-related recordings were taken from the initial 5 minutes of each task for consistency and to ensure that HRV calculations reflected equivalent states of cognitive load and stress.

HRV was operationalised using power spectral density analysis of R-R interval variability in the low (LF, 0.04–0.15Hz), and high (HF, 0.15–0.4Hz) frequency ranges. These frequency-domain measures have been explored in recent tDCS research [31–33] and are thought to provide reliable markers of sympathetic and parasympathetic activity, respectively [6]. All measures were normalised using log transformation.

### Statistical analysis

Active and sham groups were compared at baseline for similarity in age, gender, working memory, and HRV using independent samples t-tests or Fisher’s exact test where appropriate.

Accuracy scores (i.e. percentage of correct responses) and response time on the working memory task were analysed using a 2 x 3 mixed analysis of variance (ANOVA) with a between-subjects factor of Group (active tDCS, sham tDCS) and a within-subjects factor of Time (baseline, during-tDCS, post-tDCS). Greenhouse-Geisser corrections were adopted in the event of violations of Mauchly’s test of sphericity.

A linear mixed effects model was used to examine the changes in HRV over time, with baseline HRV included as a covariate. The four time points analysed in the model were rest and task-related activity in both the during-tDCS, and post-tDCS, periods (see Fig 2). Past research has identified a generalised reduction over time for HF-HRV, and a concurrent increase in LF-HRV, possibly due to mental stress and cognitive fatigue from experimentation [33–35], thus prompting the inclusion of time as a repeated factor in this analysis. Fixed factors were Group (sham tDCS, active tDCS), Task (rest, working memory task), and Period (during-tDCS, post-tDCS), in addition to the two-way interactions of Group × Task, Group × Period, Task × Period, and the three-way interaction of Group × Task × Period. Participant identity was included as a random effect.

Following both ANOVA and mixed effects model analyses post-hoc tests were conducted on simple main effects using pairwise comparisons. Statistical significance was set at p < .05.

The neurovisceral integration model predicts that increased activity of the prefrontal cortex will be associated with greater parasympathetic, and decreased sympathetic, autonomic activity as well as better working memory (see Fig 1). This is regardless of whether greater prefrontal activation occurs due to endogenous task engagement (i.e. sham group), or a combination of task engagement and additional neuronal activation from an external source (i.e. brain stimulation as in the active tDCS group). To verify this model, Pearson correlation coefficients were computed for the relationship between changes from baseline in individual working memory accuracy scores and HRV for both HF and LF bands. Correlations were performed with combined data from participants from both active and sham tDCS conditions.

## Results

Participant characteristics at baseline were well matched and showed no significant differences between active and sham groups (see Table 1).

**Table 1:** Demographic information. Working memory (WM) performance calculated using the percentage and response time (RT) of correct responses; LF (ln(ms^2^)), low-frequency (log transformed); HF (ln(ms^2^)), high-frequency (log transformed).

	Sham (SD)	Active (SD)	p
Age	23.3 (3.4)	22.3 (3.8)	0.541
Gender (M/F)	(7/3)	(4/6)	0.370
Baseline values			
WM (%)	75.3 (11.5)	78.7 (10.9)	0.513
WM (RT)	800.2 (104.2)	756.7 (103.1)	0.361
LF_ln_	6.35 (0.70)	5.97 (0.52)	0.182
HF_ln_	6.12 (0.93)	6.18 (0.66)	0.886

### Blinding

Participants recruited to this study were naïve to tDCS and were only exposed to one condition (i.e. either active or sham tDCS). At the completion of the experiment participants were asked to guess whether they had received active or sham tDCS. Analysis of participant guesses using Fisher’s Exact Test revealed no significant differences in accuracy (p = 0.629).

### Effect of tDCS on working memory performance

**Fig 3:**
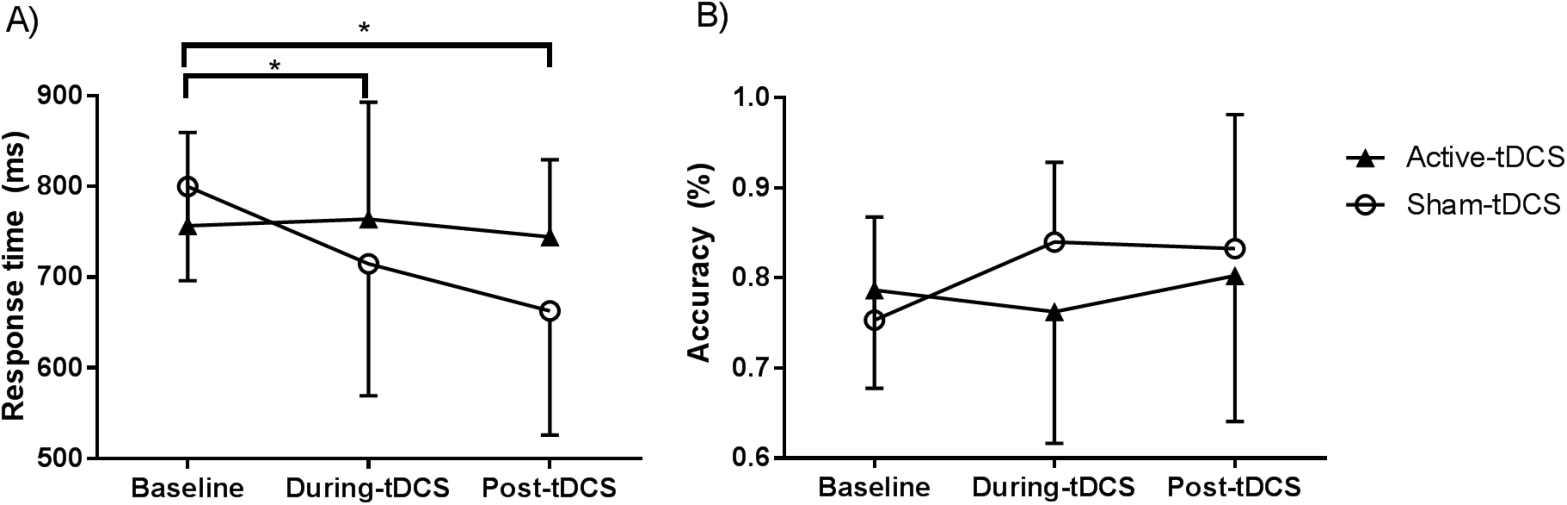
Working memory scores. (A) Participants receiving sham-tDCS improved in response time from baseline to during-tDCS and post-tDCS time points. (B) Working memory accuracy scores calculated as percentage of correct responses. Error bars represent standard deviations. * p < .05.

There was no main effect of Time (F_(2,36)_ = 2.128, p = 0.134), Group (F_(1,18)_ = 0.228, p = 0.639), or Time × Group interaction (F_(2,36)_ = 2.814, p = 0.073) for working memory accuracy scores (Fig 3B).

Working memory response time scores revealed a significant main effect of Time (F_(2,36)_ = 5.265, p = 0.018), but not Group (F_(1,18)_ = 0.393, p = 0.539). The Time × Group interaction was also significant (F_(2,36)_ = 3.957, p = 0.041). Post-hoc analyses revealed improvements from baseline to during-tDCS (p = 0.028) and post-tDCS (0.002) in the sham group, but not the active group (p = 0.841 and p = 0.749 for during-tDCS and post-tDCS periods, respectively; Fig 3A).

### HRV Data

Log transformed values of HRV scores are displayed in Table 2.

**Table 2:** Observed heart rate variability values expressed in frequency-domain values. All HRV metrics were natural log transformed. LF_ln_ (ms^2^), low-frequency; HF_ln_ (ms^2^), high-frequency.

	Sham tDCS				Active tDCS			
	LF_ln_		HF_ln_		LF_ln_		HF_ln_	
	mean	SD	mean	SD	mean	SD	mean	SD
Baseline	6.352	0.704	6.124	0.927	5.967	0.519	6.177	0.661
During-tDCS								
Rest	6.555	0.685	5.893	0.958	6.372	0.694	6.330	0.670
Task	5.916	0.939	5.580	1.442	6.580	0.932	6.035	1.053
Post-tDCS								
Rest	6.476	0.591	5.740	0.892	6.360	0.728	6.122	0.814
Task	6.798	0.818	5.454	1.284	6.609	0.773	5.966	0.812

**Fig 4:**
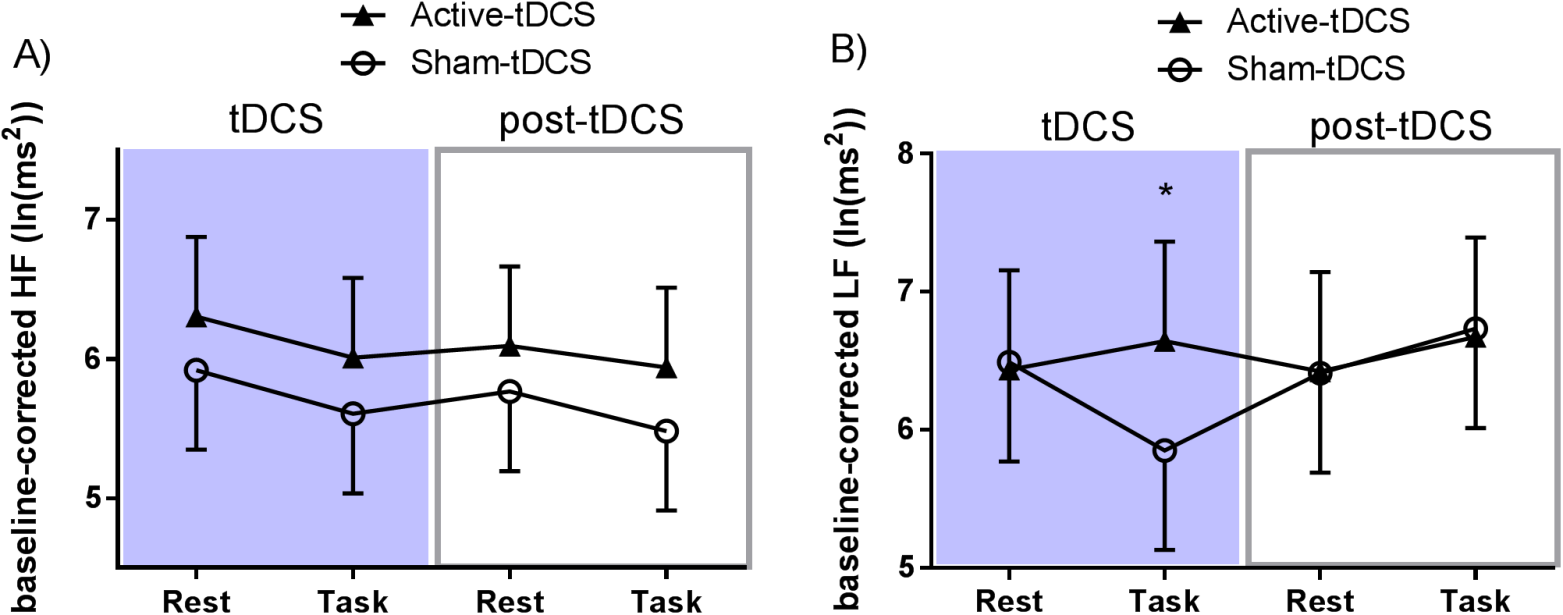
Results of baseline-corrected electrocardiogram HRV analyses using estimated marginal means from mixed effects model analysis. (A) High frequency (HF) power. (B) Low frequency (LF) power. Error bars represent standard deviations. * p < .05.

Linear mixed effects model analysis of HF-HRV showed no effect of Period (F_(1,60)_ = 1.572; p = 0.215), however, there was a significant fixed effect of Group (F_(1,20)_ = 5.447; p = 0.030; Cohen’s d = 1.04), as well of Task (F_(1,60)_ = 5.621; p = 0.021; Cohen’s d = 0.75). HF was greater overall for the active-tDCS group compared to sham-tDCS, and rest compared to task periods (see Fig 4A). There were no significant interactions.

There were no main fixed effects in the LF band for Group (F_(1,20)_ = 0.487; p = 0.493), Task (F_(1,60)_ = 0.078; p = 0.781), or Period (F_(1,60)_ = 2.708; p = 0.105); however, there was a marginally significant two-way interaction effect between Task and Period (F_(1,60)_ = 4.052; p = 0.049), as well as a non-significant three-way interaction effect (F_(1,60)_ = 3.418; p = 0.069).

A post hoc test of rest vs. task-related activity in the sham condition was used to examine the effect of a working memory task on HRV. This did not reach significance for both LF-HRV (p = 0.372) and HF-HRV (p = 0.060).

Pairwise comparisons were conducted comparing active and sham tDCS for 1) the during-tDCS period at rest to assess the effect of tDCS alone; 2) the post-tDCS period at rest to test whether the effects of tDCS persist in the after-effect period; and 3) task-related activity during-tDCS to examine combined effects of task and stimulation. None of these outcomes were significant for HF-HRV (tDCS-alone: p = 0.138, after-effect of tDCS: p = 0.204, tDCS with task: p = 0.121). Similarly, there was no effect on LF-HRV for both tDCS alone (p = 0.867) and for tDCS after-effects (p = 0.969). However, there was a significant difference between sham and active conditions in task-related LF power during-tDCS, showing a combined effect of task with tDCS (p = 0.019) – see Fig 4B.

### Working memory outcomes and HRV data

**Fig 5:**
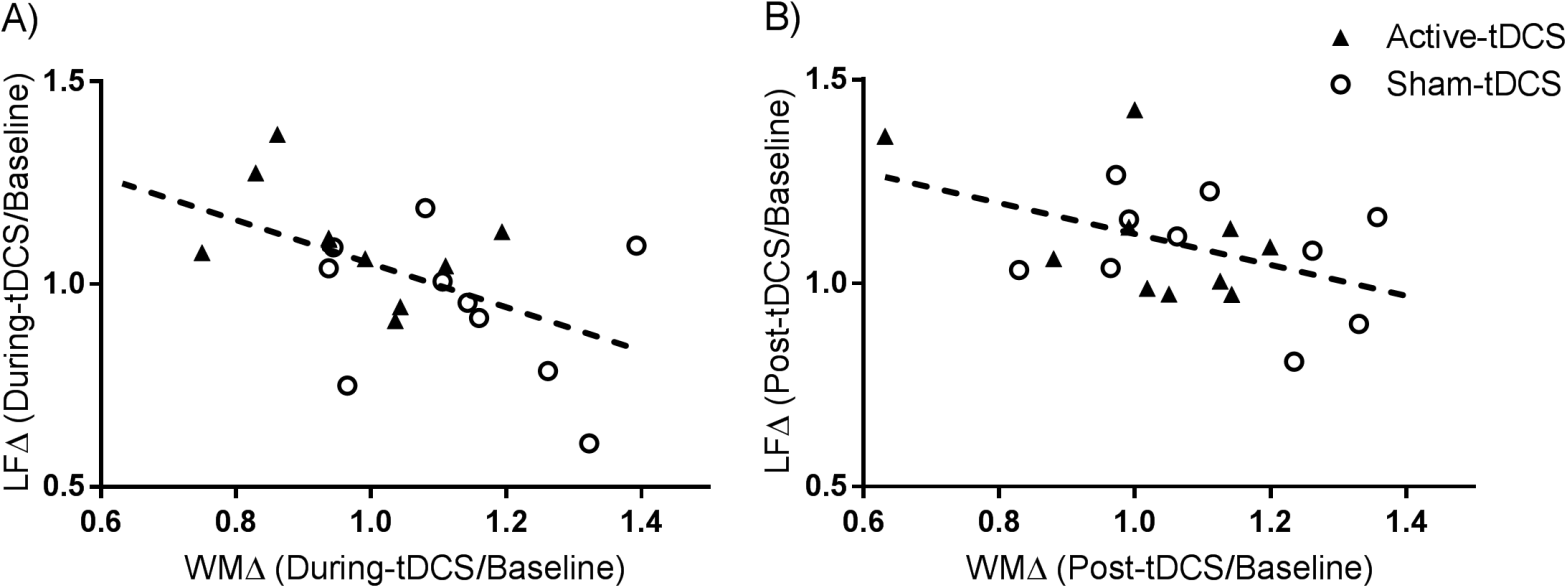
Correlation between the change in working memory accuracy and change in LF power. (A) change from baseline to during-tDCS task period; and (B) change from baseline to post-tDCS task period.

Pearson correlation coefficients for the relationship between working memory performance and HF-HRV were not significant for both during-tDCS (r = -0.09, p = 0.717), and post-tDCS (r = -0.13, p = 0.588) task periods. For LF-HRV there was a significant negative correlation with working memory accuracy in the during-tDCS task period (r = -0.50; p = 0.024), however, this association did not quite reach significance in the post-tDCS task period (r = -0.44; p = 0.050) – see Fig 5.

## Discussion

This study investigated the combined effects of prefrontal tDCS and a cognitive task known to activate the prefrontal cortex on heart rate variability in healthy participants. We hypothesised an increase in parasympathetic, and decrease in sympathetic, activity with tDCS onset, which would persist beyond stimulation, and correlate with performance on a working memory task. Differential effects were found on HRV for resting-state and task-related activity. Active tDCS resulted in increased HF power compared to sham, consistent with increased vagal tone with prefrontal stimulation, thought to reflect parasympathetic disinhibition. During task performance, tDCS both inhibited working memory performance and suppressed adaptive modulation of LF power, which was evident during sham stimulation, in response to a cognitively demanding task. Finally, changes in LF power from baseline were inversely associated with working memory performance across both conditions.

Unexpectedly, in our study working memory performance significantly improved in terms of faster response time under sham stimulation but not in the group receiving active tDCS. We think the most likely interpretation of this finding is that participants receiving sham tDCS improved due to a practice effect, whereas in those receiving active tDCS stimulation may have hindered the practice effect, i.e. faster responding on the working memory task.

In addition to impeding performance on the working memory task, active tDCS also resulted in a concurrent increase in parasympathetic tone. Interestingly, this disagrees with our initial hypothesis, which predicted better cognitive performance to coincide with greater HF-HRV as both are correlates of prefrontal activation [11,12]. Meta-analyses of prefrontal tDCS effects on working memory found that tDCS improved response time, but not accuracy, in healthy participants in agreement with our findings [19,20]. Using a similar working memory task (i.e. 3-back task), past studies have shown increased task performance accuracy compared to sham following anodal tDCS to the left DLPFC [24,36]. These studies differed slightly from our experiment design in that they used a lower current intensity (1mA), and a montage placing the cathode on the contralateral supraorbital region (Fp2) instead of the right DLPFC, which may partly explain the opposite findings. Additionally, our electrodes were approximately half the area of the ‘standard’ 5 Χ 7cm in use in the majority of tDCS experiments, resulting in a much greater current density at the stimulation site. Interestingly, a recent meta-analysis of prefrontal tDCS cognitive effects suggested that greater current density is a positive predictor of improved working memory performance outcomes in healthy participants [37], as examined in studies with a range of current densities of 0.03–0.08 mA/cm^2^. However, this study used a current density of 0.125 mA/cm^2^, raising the possibility that the dose-response curve may have an inverted-U shape, and that higher current densities as used in this study may impair function in healthy volunteers.

The observed variations in HF-HRV can be interpreted as the aggregate outcome of linearly additive effects operating on different time scales; cumulative mental stress and fatigue due to the experimentation process [33,34]; task-related cardioacceleration resulting in reduction to both parasympathetically and sympathetically mediated HRV [38–40]; and finally, preferential activation of key brain regions (e.g. excitatory tDCS to the prefrontal cortex) altering HRV output in line with predictions from the neurovisceral integration model.

Our observation of increased HF-HRV, which occurred immediately during tDCS of the prefrontal cortex and endured beyond the end of stimulation, seems to accord with existing literature. Montenegro et al. [41] found greater HF-HRV and reduced LF-HRV values following left temporal tDCS, indicating enhancement of cardiac parasympathetic activity and reduced sympathetic activity, respectively. Similarly, anodal stimulation of the left insular cortex in healthy elderly volunteers significantly raised HF-HRV and altered the sympatho-vagal balance (calculated as a ratio of LF/HF) in favour of greater parasympathetic tone [42]. Adopting a bifrontal tDCS montage comparable to the one employed in the current study, Brunoni et al. [2] observed increased vagal activity during visual presentation of negative stimuli in a test of emotional arousal. Lastly, Petrocchi et al. [43] observed that a single 15 minute session of 2mA tDCS applied to the left temporal lobe was able to increase vagally mediated HF-HRV and produce a concurrent soothing positive affect. These findings are in agreement with the neurovisceral integration model, which proposes shared neural circuitry, originating from the prefrontal cortex, that both regulates the parasympathetic and sympathetic branches of the autonomic nervous system, and modulates cognitive activity [5]. Furthermore, the left hemisphere has been linked to preferential regulation of vagal sinus arrhythmia [44], and in particular the medial prefrontal cortex has been correlated with the vagal component of HRV, suggesting an inhibitory role on autonomic regulation [7]. Thus, our findings can be interpreted as preferential activation of parasympathetic neural circuitry via increased activity of the left DLPFC, an important node in the prefrontal, using excitatory anodal tDCS. However, it is important to note that a clear picture regarding the effects of tDCS on HRV is yet to emerge, largely due to the scarcity of tDCS studies assessing HRV as well as the high degree of heterogeneity in experiment designs [45,46]. For example, two studies have reported null findings, showing no difference in HRV between sham and active tDCS in healthy participants at rest [33,35], while another noted an increase in LF-HRV following stimulation in direct contrast to our results [32].

We observed significant reductions in HF-HRV during working memory task conditions compared to rest. This represents a robust finding in the HRV literature, for which there is considerable experimental supporting evidence [47–49]. Increases in respiration rate (both amplitude and frequency), heart rate, and blood pressure, which result in a corresponding decrease in HRV, are taken as symptomatic of an altered physiological state in response to task and are generally attributed to mental stress responses [50]. Thus, replication of these previous findings lends further credibility to our data.

Contrary to expectation, we were unable to find evidence of an association between working memory improvement and HF-HRV, though an inverse relationship was found with LF-HRV. Previous studies have developed research protocols using HRV measures as an independent variable by dividing participants at baseline into either high-HRV or low-HRV groups. The outcomes of these studies provide evidence linking cardiac parasympathetic activity to working memory. For example, Hansen et al. [9] showed that participants with a higher root mean square of successive interbeat interval differences (RMSSD; thought to reflect vagal modulation) at baseline had greater accuracy on a working memory task, and had a reduction in vagally mediated HRV during task presentation. This is consistent with our results, which found a significant reduction in HF-HRV during task presentation compared to baseline. However, these changes in HF-HRV were not associated with working memory performance. Beyond the tDCS literature, improvement of executive performance on a Stroop task has been associated with an autonomic reduction in HF-HRV (0.12–0.4Hz band) and a corresponding decrease in the heart period [51].

Studies of the effects of cognitive load on HRV have shown similar results. An evaluation of HRV during a cognitively demanding working memory task, such as the 3-back task, revealed that the amplitude of the LF component (0.1Hz) decreased as the working memory load increased [21]. Comparably, spectral analyses of 10 healthy male participants showed a reduction in power in the LF component (0.06–0.12Hz band) with increased task-related processing demands [52]. Likewise, physiological data in combat pilots showed a tendency for low frequency power to decrease during high levels of information load [53]. Our finding of a medium-to-strong inverse correlation between changes in LF-HRV and changes in working memory performance is, to the best of our knowledge, a novel finding. This association may reflect a link between sympatho-adrenal system suppression due to the cognitive stress brought on by a challenging memory task and the resulting impact such exertions of mental effort have on performance outcomes. Independent lines of research suggest that moderate-high levels of mental stress both increase LF-HRV [54], and decrease working memory performance [55]. These results tentatively indicate that LF-HRV may be a measure of task-related engagement of the prefrontal cortex, revealing a shift in sympatho-vagal balance to incorporate a higher degree of sympathetic tone. Thus, participants who improved in working memory performance tended to show reduction in sympathetic tone (LF-HRV power) compared to baseline, whereas those who did not improve showed an increase in sympathetic activity. Additional research using larger samples is required to understand this relationship in greater detail and determine whether these findings are robust.

Speculatively, HF-HRV may primarily reflect tDCS-induced alterations to neural circuitry shared between the parasympathetic cardiac control network and the fronto-parietal network, which includes the DLPFC. LF-HRV, however, may predominantly measure the intersection between the sympathetic cardiac control network and cognitive control network required for working memory, which also includes the DLPFC [56]. In agreement with this interpretation is supporting evidence that the sympathetic and parasympathetic branches of the autonomic nervous system have similar, but distinct, neural pathways, although further research is required to understand these circuits in detail [5,44].

### Limitations

Although previous studies have obtained significant findings with similar sample sizes, these have used within-subject designs to increase statistical power [2,41]. Therefore, our study is limited by its small sample size, and is likely underpowered to probe the more complex interactions of tDCS and HRV. However, agreement with past research suggests a consistent effect.

The current experiment design does not allow us to specify whether correlations between LF-HRV and WM performance are the result of generalised alterations to prefrontal cortex functioning or due to other brain regions involved in WM processing. Additional research utilising control tasks that also engage the prefrontal cortex, and/or engage the prefrontal cortex to a lesser extent than during WM processing, is therefore required to further explore this association.

Finally, the study design was cognitively demanding, which therefore may have resulted in excessive cognitive stress. This effect may have carried over into resting-state recordings of HRV and thus minimised ability to infer statistical significance. However, cognitive load was equivalent in both groups, therefore comparisons should yield valid differences. Future studies should allow additional time between tasks for participants’ HRV to return to baseline.

## Conclusions

Measures of HRV present as potential indices for activation of cortico-subcortical neural circuitry following activation of the prefrontal cortex both during vagally dominated periods of resting-state activity and sympatho-adrenal mediated task-related activity. This network is shared for both cognitive processes, as well as regulation of cardiovascular control via changes to sympatho-vagal tone, and can be feasibly assessed using HRV in conjunction with more standardised behavioural outcomes. A frequency-domain HRV measure in the HF band, a marker of parasympathetic activity, was able to detect increased vagal activity both during, and in the 15 minutes following tDCS. Additionally, power in the LF band was found to correlate with changes in cognitive functioning, indicating an association between PFC activity and the sympathetic branch of the autonomic nervous system.

Given the ease of HRV data collection (requiring as few as two electrodes), studies of prefrontal cortex stimulation would benefit from collection of ECG data as it provides additional insight into distributed effects of tDCS from propagation through pathways connecting the DLPFC. Future tDCS research might benefit from concurrent use of HRV in patient populations (as in Brunoni et al. [31]) in conjunction with other physiological measures, such as electroencephalography (EEG) and galvanic skin response (GSR), and additional probes of cognitive functioning to develop a multimodal model of the efficacy of tDCS interventions.
